# Effects of *Saccharomyces cerevisiae* and *Cyberlindnera fabianii* Inoculation on Rice-Flavor Baijiu Fermentation

**DOI:** 10.3390/foods13193175

**Published:** 2024-10-06

**Authors:** Jinglong Liang, Haishan Yuan, Yongtao Fei, Hong Wang, Chunyun Qu, Weidong Bai, Gongliang Liu

**Affiliations:** 1 Guangdong Provincial Key Laboratory of Lingnan Specialty Food Science and Technology, College of Light Industry and Food Technology, Zhongkai University of Agriculture and Engineering, Guangzhou 510225, China; jinglong_liang@zhku.edu.cn (J.L.); yhs576824367@126.com (H.Y.); yongtaofei@zhku.edu.cn (Y.F.); whongaq@163.com (H.W.); quchunyun1990@126.com (C.Q.); whitebai2001@163.com (W.B.); 2 Key Laboratory of Green Processing and Intelligent Manufacturing of Lingnan Specialty Food, Ministry of Agriculture and Rural Affairs, Zhongkai University of Agriculture and Engineering, Guangzhou 510225, China; 3 Academy of Contemporary Agricultural Engineering Innovations, Zhongkai University of Agriculture and Engineering, Guangzhou 510225, China

**Keywords:** rice-flavor baijiu, ester-producing yeast, inoculated fermentation, metagenomic analysis

## Abstract

Rice-flavor baijiu is a distilled Chinese spirit prepared from Xiaoqu culture. However, its dull taste may be a market limitation. In order to enhance the flavor profile of rice-flavor baijiu, two ester-producing yeast strains (*Saccharomyces cerevisiae* and *Cyberlindnera fabianii*) were inoculated for fermentation. At the end of the fermentation, the total alcohol and ester contents had also increased by 43.3% and 29.8%, respectively, and the number of ester species had increased by eight. Additionally, eleven flavor substances had significant contributions in the inoculated fermentation process, including several different esters and alcohols. A macrogenomic analysis revealed that the majority of the gene abundances associated with the alcohol, acid, and ester pathways were elevated by the third day of inoculated fermentation, and greater abundances of *Saccharomyces cerevisiae*, *Cyberlindnera fabianii*, *Lichtheimia ramosa*, *Rhizopus delemar*, and *Rhizopus oryzaefive,* annotated with these genes, were observed from either the pre-fermentation stage or post-fermentation stage. The results demonstrate that two added strains are associated with an increase in the content of the flavor substances. These findings may prove beneficial in enhancing the quality of rice-flavor baijiu through using inoculated fermentation with ester-producing yeast.

## 1. Introduction

Rice-flavor is one of the four dominant aromas in Chinese baijiu. Rice-flavor baijiu is produced through the fermentation of rice using a semi-solid process involving the addition of Xiaoqu starter [1]. The main flavor substances present in rice-flavor baijiu are esters, including ethyl acetate, ethyl lactate, and phenylethanol [2]. These impart a sweet flavor and clean taste to the spirit. Currently, some consumers perceive the main drawbacks of rice-flavor baijiu to be its unexciting and smooth flavor, the absence of a luxurious and subtle aroma, and the esters are lower and less diverse, all of which significantly impact the quality of baijiu [3]. The growth and metabolic processes of the microoraganisms in the fermentation starter exert a profound influence on the flavor of baijiu [4]. The dominant bacterial genera in the starter culture are *Lactobacillus*, *Welchiella*, *Lactococcus*, and *Acetobacter*, and the dominant fungal genera are *Rhizopus*, *Pichia*, *Saccharomyces*, and *Issatchenkia* [5,6]. The production of the traditional Xiaoqu starter is conducted in an open, natural setting, utilizing a limited range of raw materials. This approach has resulted in a starter with relatively decreased flavor-producing microbial populations [7]. Over the past few years, there has been a notable rise in the discovery of microoraganisms from traditional fermentation processes that enhance the flavors in baijiu fermentation, for example, *Wickerhamomyces anomalus*, *Saccharomyces cerevisiae*, *Hyphopichia burtonii*, *Clavispora lusitaniae*, *Saccharomycopsis fibuligera*, *Bacillus velezensis*, *Bacillus subtilis*, and other ester-producing strains. The inoculation of functional microoraganisms into the baijiu fermentation has a positive effect on the microbiota, enzymatic activity, and metabolite composition [8]. The utilization of enhanced cultures can result in an enhancement of the production of the flavor substances and can facilitate the production of beneficial effects in a variety of baijiu [9,10,11,12,13,14,15]. Acetates are important flavor substances, the content of which is an important index to distinguish the quality of rice-flavor baijiu. Acetates were synthesized from the alcohol acyltransferase pathway, alcohol dehydrogenase pathway, and esterification pathway of microoraganisms [3]. Ester-producing yeasts, including *Saccharomyces cerevisiae*, *Clavispora lusitaniae*, *Cyberlindnera fabianii*, and others, possess these pathways and are closely related to the synthesis of various ester substances and precursors in alcoholic beverages [16,17,18]. The inoculation of ester-producing yeast into the fermentation process of Xiaoqu baijiu has been demonstrated to have a positive effect on the production of esters [12,15]. The findings of these studies provide new insights into the means of enhancing the quality of rice-flavor baijiu.

However, most of the strains used for inoculated fermentation are derived from the homologous starter, and there may be differences between the inoculated fermentations of the same strain with a different starter. Thus, further exploration of the role regarding the inoculation of rice-flavor baijiu fermentation with flavor-producing strains is needed. The rapid advancement of molecular biology techniques has enabled researchers to extend their investigation of the microbial community structure in fermented foods beyond the traditional research involving separable and cultured microoraganisms. The application of high-throughput sequencing technology in microbial population studies offers a number of advantages over the traditional approaches. These include insights into the composition, distribution, and dynamic changes in the microbial populations. Metagenomic sequencing technology provides rich genetic information of microbial species and improves the detection efficiency and accuracy of the results [19,20].

A microbial combination comprising *Saccharomyces cerevisiae* and *Cyberlindnera fabianii*, which exhibits a robust capacity for acetate production within the Xiaoqu baijiu environment, was previously obtained [21]. The objective of this study is to brew rice-flavor baijiu using two yeast strains inoculated into a fermentation in order to improve the flavor substances. The changes in the flavor substances and key gene abundance during the fermentation were examined by utilizing headspace solid-phase microextraction (HS-SPME) coupled with gas chromatography–mass spectrometry (GC–MS) and metagenomic sequencing technology, respectively. The generated data revealed the difference regarding the enhanced inoculation on the key flavor substances and key pathways during fermentation, which provide a reference for understanding the role of the enhancement of the flavor substances in rice-flavor baijiu via the inoculated fermentation with an ester-producing yeast.

## 2. Materials and Methods

### 2.1. Fermentation and Sampling

**Figure 1:**
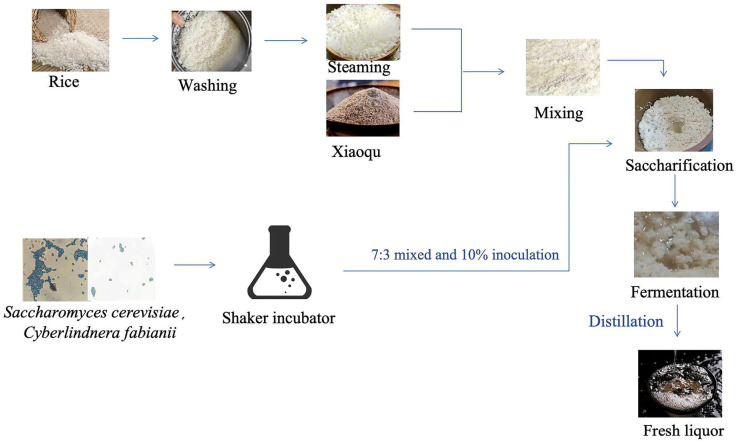
The process of two strains’ inoculation on rice-flavor baijiu fermentation.

*Saccharomyces cerevisiae* (GDMCC 63610) and *Cyberlindnera fabianii* (GDMCC 63612), which were isolated from different Xiaoqu starters, can produce a high yield of esters. Rice material was purchased from a local market. Xiaoqu starter was donated by a baijiu factory in Guangdong Province, China. Figure 1 shows the fermentation process for rice-flavor baijiu undertaken in this study. In the normal fermentation process, deemed CK in this study, the rice was washed twice, soaked in water, and then steamed for 15 min. Following cooling of the steamed rice mixture to less than 30 °C, the 250 g of mixture was combined with 250 g of the 0.4% (*w*/*w*) Xiaoqu starter, which had been stirred until homogeneous. Subsequently, saccharification of the mixture occurred at 34 °C for a period of 40 h. The final stage involved the fermentation with addition of 300 mL of sterile water at 28 °C for a period of 15 days. The inoculated fermentation process was conducted according to previous research [21], termed SY in this study, and *Saccharomyces cerevisiae* and *Cyberlindnera fabianii* were activated in a shaking incubator, mixed in a 7:3 ratio, and then inoculated at 10% (mL/g) in a fermentation jar. Three biological replicates were performed. Samples comprising a combination of liquids and solids were collected daily for the next 15 days for HS-SPME-GC–MS. In addition, samples from 3d and 11d of fermentation were used for sequencing. The samples were stored at a temperature of -80 °C until subsequent analyses were conducted.

### 2.2. Analysis of the Flavor Substances

The volatile flavor substances were determined using an Agilent 6890A-5973N (Agilent, Santa Clara, CA, USA) with an Agilent DB-WAX UI column (30 m × 0.25 mm × 0.25 μm) [22]. The samples were subjected to centrifugation in order to obtain the supernatant for analysis. An internal standard was prepared with n-pentyl acetate in ethanol solution. Next, supernatant was mixed with NaCl and amyl acetate internal standard solution and preheated at 45 °C for 10 min. Subsequently, the mixture was extracted by activated extraction needle for 50 min. The carrier gas was helium. The flow rate was 1 mL/min. The injection port temperature was 250 °C. The split ratio was 20:1. The detection time was 5 min. The column temperature program in GC was 40 °C for 2 min; heat to 100 °C at a rate of 5 °C/min and hold for 10 min; and heat to 200 °C at a rate of 10 °C/min and hold for 10 min. The MS source temperature was 230 °C. The MS quadrupole was 150 °C. The MS was operated in electron impact mode with an electron energy of 70 eV, a full scan range, and mass scanning range (*m*/*z*) of 50–550. All experiments were conducted in triplicate.

### 2.3. Total DNA Extraction and Sequencing

A volume of 25 mL of sample was taken and subjected to centrifugation at 12,000 r/min for 15 min in a centrifuge tube to remove the supernatant. An appropriate amount of sediment sample was taken and grinded thoroughly in a liquid nitrogen environment. Approximately 100 mg of powder was transferred to a pre-cooled centrifuge tube for DNA extraction. Total DNA was extracted using a HiPure DNA Kit (Magen, Guangzhou, China) according to the manufacturer’s instructions. The quality of the genomic DNA was measured by NanoDrop microspectrophotometer (Thermo Fisher Scientific, Waltham, MA, USA) and agarose gel electrophoresis. DNA fragments were modified using the NEBNext^®^ Ultra^™^ DNA Library Prep Kit for Illumina^®^ (NEB, Ipswich, MA, USA) according to the manufacturer’s protocol. Next, DNA fragments were used as template of PCR for 300 to 400 bp in length of DNA enrichment. The libraries were analyzed for size distribution using the Agilent 2100 (Agilent, Santa Clara, CA, USA). The metagenomic libraries were sequenced on an Illumina HiSeq 2500 sequencing platform at Guangzhou Gene Denovo Co., Ltd. (Guangzhou, China). The original data of metagenomic were submitted to the Sequence Read Archive (SRA) database of the National Center for Biotechnology Information (NCBI). The accession number is PRJNA1067078.

### 2.4. Bioinformatic Analysis and Function Annotations

Raw data were filtered using FASTP (version 0.18.0) [23]. The filtered clean data were assembled into effective reads using MEGAHIT software (version 1.1.2) [24]. The genes of contigs > 500 bp were predicted and clustered using MetaGeneMark (version 3.38) [25] and CD-HIT software (version 4.6) [26]. The re-aligned clean reads rearrange to the initial non-redundant gene set using Bowtie2 (version 2.2.5) [27]. Based on the comparison results, the final set of genes for subsequent analysis was obtained using PathoScope software (version 2.0). The relative abundance of genes was calculated from the number of reads allocated to genes, gene length, and sequencing depth. The unigenes were obtained by comparative analysis of DIAMOND software (version 2.1.1) from the NR (Non-redundant Protein), KEGG (Kyoto Encyclopedia of Genes and Genomes), eggNOG (Evolutionary genetics of genes: Non-supervised Orthologous Groups), and CAZy (Carbohydrate Active enZYmes) databases. Microbial community functions in the samples were analyzed in accordance with the comparison results. Systematic analysis and comparison of different groups were conducted according to the abundance information from different databases.

### 2.5. Construction of the Flavor Substance Metabolic Network

Based on the results of metagenomic species and function annotation, KEGG was used to construct the metabolism of the key flavor substances generated by microoraganisms during the fermentation of two groups (CK and SY). Heat maps were drawn using species annotation and function annotation. Information for related enzymes in the metabolic pathway was clarified, and the related enzymes and microoraganisms with gene ID through the NR database were connected. Finally, the metabolic interconnection between the formation of key flavor substances and microoraganisms in two groups was established.

### 2.6. Statistical Analysis

Principal component analysis (PCA) was performed using SIMCA software (version 14.1). The statistical analyses were performed using IBM SPSS version 22 and Origin 2019 (OriginLab Co., Northampton, MA, USA). Comparisons of the flavor substances’ contents were analyzed by *t*-test with *p* < 0.05 as the significant difference. The analyses of gene abundances were performed using Morpheus (https://software.broadinstitute.org/morpheus, accessed on 20 August 2024).

## 3. Results and Discussion

### 3.1. Differences in Flavor Substances between Two Fermentation Processes

**Figure 2:**
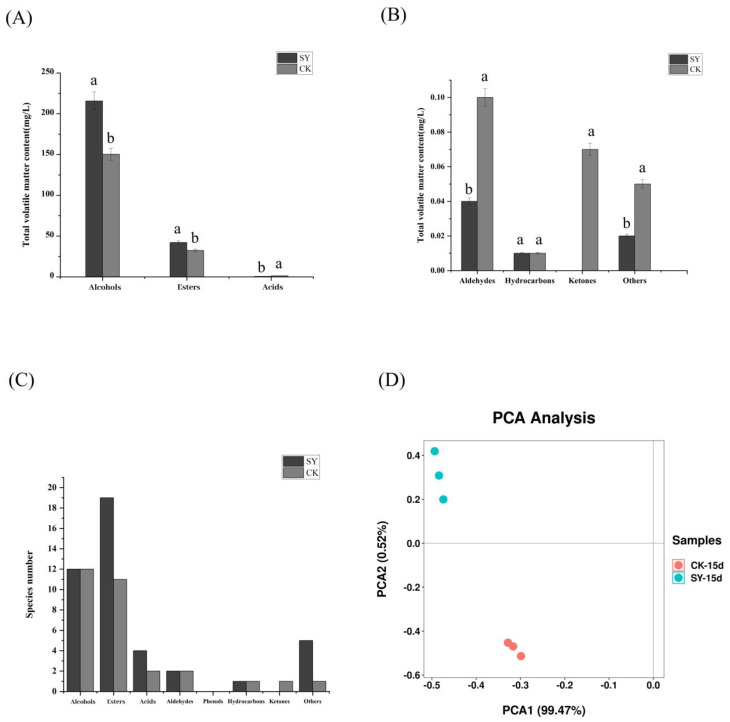
Volatile flavor substances in SY and CK upon completion of fermentation. (**A**,**B**) Total concentration; (**C**) species number; (**D**) principal component analysis. Note: Columns with different letters indicate significant differences (*p* < 0.05).

The dynamic changes in the volatile substances in the two groups (CK and SY) were shown in Figures S1 and S2. The analysis revealed a total of 151 different flavor substances. These included twenty-three alcohols, fifty-one esters, ten acids, ten aldehydes, three ketones, seven phenols, twenty-one hydrocarbons, and twenty-six other compounds. The number and content of volatile flavor substances in SY were more than in CK (Figure 2A–C). The proportions of alcohols and esters in SY had also increased by 43.3% and 29.8%, respectively, and the number of ester species had increased by eight. The changes in the esters and alcohols were consistent with the results of Xiaoqu bioenhanced fermentation with ester-producing yeast [12,15]. Alcohols were reported to be important for the sense and quality of Xiaoqu baijiu and were mainly derived from microbial fermentation and amino acid metabolism, as well as being the precursor to the formation of esters [28]. A significant disparity in alcohol content was observed between the SY and the CK samples. This evidence indicates that the microbial growth, reproduction, and metabolic activities were more rapid in SY than in CK. Esters represent the most significant class of aroma compounds, exhibiting fruity and floral aromas. They play a pivotal role in the flavor of Xiaoqu baijiu [29]. The number of species and total content of esters in SY were higher than those in CK. Acids are also crucial flavor substances in baijiu, which can increase flavor and reduce irritation. The content of acids in SY was lower than in CK. A plausible reason for this difference is that some acids as precursors were converted into esters [30]. The PCA of the volatile flavor substances is shown in Figure 2D; the cumulative variance contribution of PC1 was 99.47% and PC2 was 99.72%, demonstrating that the PCA separation model was effective. The distribution between the samples of the CK and SY groups was more dispersed, indicating that the composition of the volatile flavor substances between CK and SY was different upon the completion of the fermentation.

According to the typical flavor properties and key odorants in baijiu [31], there were sixteen compounds with Odor Activity Values (OAVs) > 1 during the whole fermentation process (Figure S3), including five alcohols, nine esters, and one hydrocarbon. By the end of the fermentation, eleven flavor substances’ production in SY were significantly higher than those in CK (Table 1). Most of these volatile flavor compounds with OAV > 1 had various aromas, among which ethyl acetate and β-phenylethanol are the main aroma-presenting substances in rice-flavor baijiu [32]. The alteration of ethyl acetate was found to be in accordance with the outcomes of the inoculation of the baiju fermentation with *Wickerhamomyces anomalus* and *Saccharomyces cerevisiae* [9]. The higher contents of β-phenylethanol and phenylethyl acetate contribute to an increased honey-sweet aroma. The higher contents of isoamyl alcohol, ethyl acetate, ethyl caproate, octanoic acid ethyl ester, decanoic acid ethyl ester, dodecanoic acid ethyl ester, and tetradecanoic acid ethyl ester contribute to an increased fruity aroma. The higher contents of isoamyl alcohol, β-phenylethanol, octanoic acid ethyl ester, decanoic acid ethyl ester, and phenylethyl acetate contribute to an increased floral aroma. The higher contents of isobutanol, isoamyl alcohol, 2,3-butanediol, dodecanoic acid ethyl ester, and hexadecenoic acid ethyl ester contribute to an increased oily aroma.

### 3.2. Differences in Gene Abundance of Flavor Substance Pathways between Two Fermentation Processes

In this study, the most drastic material changes occurred during days 0 to 3 of fermentation, whereas there was a stable period after day 11 of fermentation. Therefore, the metagenomic experiments incorporated samples from these two time points. The sequencing information is shown in Table S1. The number of unique genes that could be annotated to the NR database was 60,609, representing 80.45% of the total gene catalogue. The annotated information of the metabolic pathways in the two groups under level 1 classification, as derived from the KEGG, was shown in Figure S4. In the KEGG level 2 classification of metabolic pathways, 46 subcategories of metabolic pathways were annotated and are shown in Figure S5.

#### 3.2.1. Alcohol Pathways

**Figure 3:**
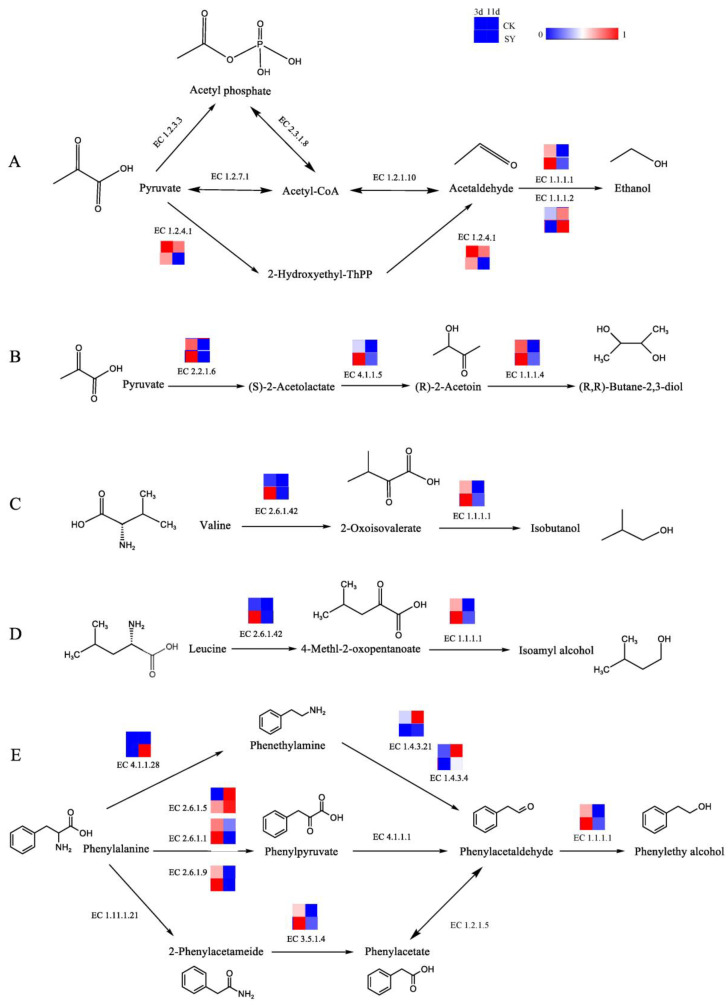
Synthesis pathways of alcohols. (**A**) Ethanol; (**B**) 2,3-butanediol; (**C**) isobutanol; (**D**) isoamyl alcohol; (**E**) phenylethyl alcohol.

Isobutanol, isoamyl alcohol, β-phenylethanol, and 2,3-butanediol are the alcohols with the highest contents in the fermentation of the two groups. In addition, the OAVs of these alcohols in SY were greater than those in CK. Ethanol is an essential component of baijiu and is primarily synthesized by transforming pyruvic acid into acetaldehyde and then reducing the acetaldehyde. The pathway of the alcohol production and related catalytic enzymes is shown in Figure 3A. In the production of acetaldehyde from pyruvate, three pathways and six enzymes are involved. According to the annotation results of the KEGG, ethanol dehydrogenase (EC 1.1.1.1 and EC 1.1.1.2) participates in the acetaldehyde reduction reaction, and its relative abundance is higher in SY during 3d and 11d of fermentation, respectively, which indicates that a substantial quantity of alcohol was generated during the fermentation. The relative gene abundance of pyruvate dehydrogenase (EC 1.2.4.1) in SY was lower than that of CK. Presumably, the flux of acetaldehyde synthesis via this pathway was lower in SY. 2,3-Butanediol has a creamy and sweet flavor. The pathway of 2,3-butanediol production and related catalytic enzymes is shown in Figure 3B. According to the annotation results of the KEGG, the relative abundances of acetolactate synthetase (EC 2.2.1.6), acetolactate (EC 4.1.1.5), and butanediol dehydrogenase (EC 1.1.1.4) were higher in SY during 3d of fermentation, respectively, which indicates a higher flux of 2,3-butanediol synthesis via this pathway in the pre-fermentation period.

The main metabolite in rice-flavor baijiu is higher alcohol, which is the flavor-presenting substance contributing to the aroma in baijiu and the precursor for synthesizing other flavor substances [33]. The Ehrlich and Harris pathways represent the two principal routes for the synthesis of higher alcohols [34]. In this study, the higher alcohols include isobutanol, isoamyl alcohol, and β-phenylethanol, which were produced during the fermentation of the two groups and had a greater OAV in SY than in CK. The three pathways for producing higher alcohols, together with the related catalytic enzymes, are illustrated in Figure 3C–E. The conversion of valine and leucine to 2-oxo-3-methylbutyric acid and 4-methyl-2-oxovaric acid, respectively, is catalyzed by branched-chain amino acid aminotransferase (EC 2.6.1.42). Then, isobutanol and isopentyl alcohol are synthesized, respectively, by EC 1.1.1.1. The relative gene abundances of EC 2.6.1.42 and EC 1.1.1.1 in SY were higher than in CK during pre-fermentation (3d). β-Phenylethanol—a compound with a honey and rose aroma—is a characteristic flavor substance in rice-flavor baijiu and can be synthesized from phenylalanine. Phenylalanine is capable of undergoing a chemical transformation, catalyzed by aromatic-L-amino-acid decarboxylase (EC 4.1.1.28), which converts it into phenylethylamine. This gene was only annotated in the late stage of fermentation (11d) in SY. Phenylethylamine undergoes oxidation to phenylacetaldehyde via the action of monoamine oxidase (EC 1.4.3.4) or primary amine oxidase (EC 1.4.3.21). This is followed by the synthesis of β-phenylethanol via the action of EC 1.1.1.1. The relative gene abundances of EC 1.4.3.4 and EC 1.4.3.21 in SY were lower than those of CK. Presumably, the flux of the phenylacetaldehyde synthesis via this pathway was lower in SY. According to the annotation results of the KEGG, the relative abundances of glutamic phenylpyruvic aminotransferase (EC 2.6.1.5), glutamic-aspartic transaminase (EC 2.6.1.1), glutamic-imidazoleacetol phosphate transaminase (EC 2.6.1.9), and amidase (EC 3.5.1.4) were higher in SY during 3d of fermentation, which indicates a higher flux of phenylacetaldehyde synthesis via these pathways in the pre-fermentation period.

#### 3.2.2. Acid Pathways

**Figure 4:**
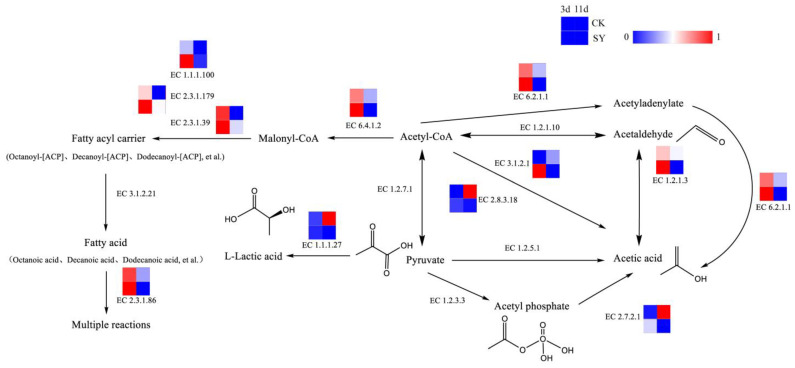
Synthesis pathways of acids.

Acids are of paramount importance in the flavor compounds of rice-flavor baijiu, acting as precursors to the formation of esters. Acetic acid, which is an important volatile acid, can be synthesized by various pathways in fermentation. The pathway of acid production and the related catalytic enzymes is shown in Figure 4. Acetic acid can be catalyzed from acetyl coenzyme A through acetyl-CoA synthetase (EC 6.2.1.1), acetyl-CoA hydrolase (EC 3.1.2.1), aldehyde dehydrogenase (EC 1.2.1.3), and succinyl-CoA: acetate CoA transferase (EC 2.8.3.18). The relative gene abundances of EC 6.2.1.1, EC 3.1.2.1, EC 1.2.1.3, and EC 2.8.3.18 in SY during the pre-fermentation period (3d) were greater than those of CK, but, in the post-fermentation stage (11d), the opposite trend occurred. The results demonstrate that a greater flux of acetic acid synthesis via these pathways is evident during the pre-fermentation period. Lactic acid, which is another significant volatile acid, serves as a precursor to the production of ethyl lactate through fermentation. The dominant pathway for lactic acid production is the pyruvate reduction reaction pathway. Pyruvate is directly generated under the catalytic reaction of L-lactate dehydrogenase (EC 1.1.1.27). The relative gene abundance of EC 1.1.1.27 in SY during the fermentation was lower than that in CK. Presumably, the flux of the L-lactic acid synthesis via this pathway was lower in SY.

Fatty acids are also important volatile acids and precursors of esters in fermentation [35] (Saerens et al., 2006). The relative gene abundances of Acetyl-CoA carboxylase (EC 6.4.1.2), [acyl-carrier-protein] S-malonyl transferase (EC 2.3.1.39), 3-oxoacyl-[acyl-carrier-protein] synthase II (EC 2.3.1.179), 3-oxoacyl-[acyl-carrier protein] reductase (EC 1.1.1.100), and fatty acid synthase (EC 2.3.1.86) in SY were higher during the pre-fermentation phase (3d), which indicates a higher flux of fatty acids and fatty-acyl-CoA synthesis via these pathways in the pre-fermentation period.

#### 3.2.3. Ester Pathways

**Figure 5:**
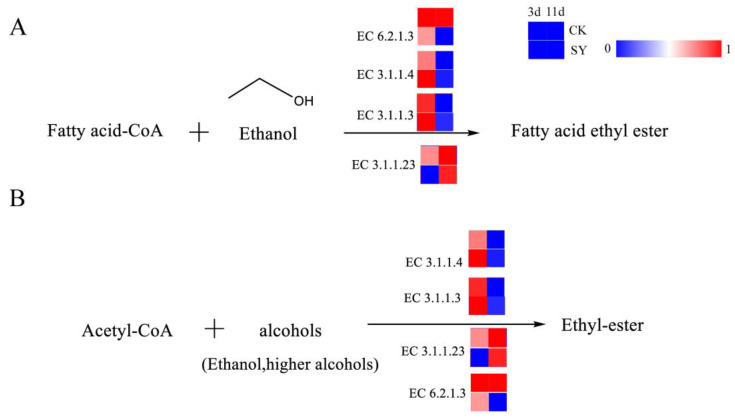
Synthesis pathways of esters. (**A**) Fatty acid ethyl ester; (**B**) Ethyl-ester.

Esters represent an essential flavoring agent during the fermentation process of rice-flavor baijiu. The analysis of the contribution of the key volatile flavor substances in Figure S3 shows that the contribution of esters to the overall flavor of rice-flavor baijiu is the most considerable factor in the two fermentation processes. These esters are subdivided into ethyl esters and acetate esters, both of which are produced through the esterification of acids and alcohols. The pathway of ester production and the related catalytic enzymes are shown in Figure 5A,B. Ethyl esters, also referred to as fatty acid ethyl esters, represent a class of chemical compounds obtained through the processes of condensation and esterification with ethanol and fatty acids. Fatty acid ethyl esters, particularly those of short- and medium-chain fatty acid ethyl esters, are flavor compounds generated via the non-oxidative pathway of ethanol metabolism in *Saccharomyces cerevisiae* and other fungi [36]. The principal metabolic pathway involved in ester production is the esterase pathway. Esterases constitute a group of enzymes that catalyze esterification reactions. They include lipases, phosphodiesterases, and ester synthases. The synthesis of fatty acid ethyl esters occurs from fatty acid acyl-CoA and ethanol by esterases. Acetate esters are also produced from higher alcohols and acetyl-CoA by esterases. In addition, the reaction between small-molecule acids such as acetic acid or lactic acid and ethanol, which forms the products ethyl acetate and ethyl lactate, can be catalyzed by esterases [37]. Of all the annotated esterases, the relative gene abundances of lipase (EC 3.1.1.3) and phospholipase (EC 3.1.1.4) in SY during the pre-fermentation were higher than those in CK, which indicates a higher flux of ester synthesis via these pathways in the pre-fermentation period.

### 3.3. Correlation Analysis between Key Pathways and Microbial Distribution during Fermentation

**Figure 6:**
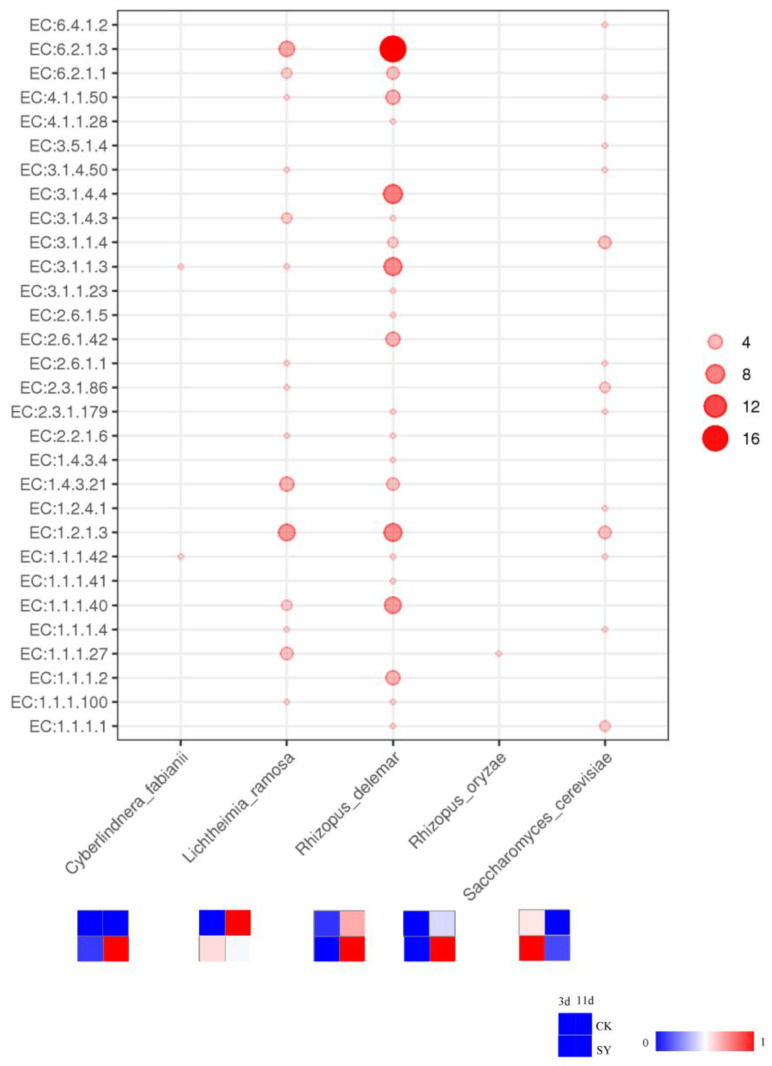
The correlation between microoraganisms and enzymes involved in different metabolic pathways.

The average relative abundances of the fungi and bacteria in the microbial community of SY were 97.899% and 2.039%, respectively, and those of CK were similar. All the gene abundances of the alcohol, acid, and ester pathways were only annotated to the fungi. The relationship between the microorganisms at the species level and the enzymes involved in the metabolic pathways is shown in Figure 6. The analysis of the microbial communities involved in the synthesis of the flavor substances was attributed to five microoraganisms in SY and CK, which were *Cyberlindnera fabianii*, *Lichtheimia ramosa*, *Rhizopus delema*, *Rhizopus oryzae*, and *Saccharomyces cerevisiae*. The relative abundance of *Cyberlindnera fabianii* was only detected in SY, and the abundance on 11d was greater than on 3d of fermentation, which indicates that added *Cyberlindnera fabianii* can grow during the fermentation and contribute to the enhancement of the flavor substances. Nevertheless, the relative abundance rank was lower, indicating that the tolerance of *Cyberlindnera fabianii* in the rice-flavor baijiu fermentation was less than that of *Saccharomyces cerevisiae*. *Rhizopus* and *Saccharomyces*, which are fungal genera, had the highest relative abundances in both groups during the fermentation. The relative abundance of *Saccharomyces cerevisiae* in SY was higher than that in CK during the fermentation, which indicates that added *Saccharomyces cerevisiae* can grow during the fermentation and contribute to the enhancement of the flavor substances. Other studies have shown that the dominant mold in Xiaoqu baijiu was also *Rhizopus* [38]. The amylase produced by *Rhizopus* can break down starch to glucose, which is a carbon source for the yeast, and these fungi also produce lipase, which facilitates the production of ester compounds in baijiu [39]. A prior study demonstrated that augmenting the concentration of *Rhizopus* in baijiu can effectively elevate the profile of esters [40]. In addition, *Rhizopus* is known to be instrumental in the fatty acid ethyl ester enhancement of baijiu [41]. The number of genes annotated to *Rhizopus delema* was higher than in other species. The relative abundances of *Rhizopus delema* and *Rhizopus oryzae* in SY during the pre-fermentation period (3d) were lower than those in CK, but, in the post-fermentation stage (11d), the opposite trend occurred. It is presumed that the increased flavor substances may be caused by the increased abundances of *Rhizopus delema* and *Rhizopus oryzae* in the post-fermentation stage. *Lichtheimia*, which is a genus of fungi, has strong heat resistance and saccharification ability [42]. The relative abundance of *Lichtheimia ramosa* in SY was greater than that in CK in the pre-fermentation period (3d), and then it decreased marginally in the later stage of fermentation, with a relative abundance lower than that of CK. It is presumed that the increased flavor substances may be caused by the increased abundance of *Lichtheimia ramosa* in the pre-fermentation stage.

Thus, it was hypothesized that these microoraganisms may be promoted during different fermentation stages by the inoculation of the two ester-producing yeast strains, and this resulted in a higher content of the key volatile flavor substances in SY compared with those in CK. Further research should distinguish and validate the roles of another three microoraganisms mentioned above in rice-flavor baijiu. Additionally, an investigation will be conducted to ascertain the reasons that the added *Cyberlindnera fabianii* cannot grow better via tolerance analysis of the strains in the environment of rice-flavor baijiu fermentation.

## 4. Conclusions

The inoculation of *Saccharomyces cerevisiae* and *Cyberlindnera fabianii* into a rice-flavor baijiu fermentation can enhance the flavor substances’ content. At the end of the fermentation, the total alcohol and ester contents had also increased by 43.3% and 29.8%, respectively, and the number of ester species had increased by eight. Additionally, eleven flavor substances had marked contributions in the inoculated fermentation process, including ethyl myristate, 2,3-butanediol, ethyl caprylate, ethyl caprate, ethyl acetate, ethyl laurate, isobutanol, phenethyl acetate, isoamyl alcohol, β-phenylethanol, and ethyl palmitate. The resulting changes in the flavor substances in the baijiu are related to greater abundances of *Saccharomyces cerevisiae*, *Cyberlindnera fabianii*, *Lichtheimia ramosa*, *Rhizopus delemar*, and *Rhizopus oryzaefive,* as observed from either the pre-fermentation stage or post-fermentation stage. The generated data revealed the difference in the enhanced inoculation regarding the key flavor substances and key pathways during fermentation, which provide a reference for understanding the role of the enhancement of the flavor substances in rice-flavor baijiu by the inoculated fermentation with ester-producing yeast. At the same time, a potential method was provided for improving the flavor substances of rice-flavor baijiu.

## Figures and Tables

**Table 1:** The volatile compounds (OAV > 1) upon completion of fermentation.

Number	Substance	Flavor Description [31]	Titer in SY (μg/L)	Titer in CK (μg/L)
A3	Isobutanol	Alcohol and nail polish aromas	14,042 ± 341 ^a^	8371 ± 1067 ^b^
A4	Isoamyl alcohol	Bitter almond, fruity, and floral aromas	39,435 ± 388 ^a^	37,331 ± 126 ^b^
A17	2,3-Butanediol	Butter and cream aromas	680 ± 11 ^a^	319 ± 54 ^b^
A20	octanol	Rose and fruity aromas	N.D.	N.D.
A22	β-Phenylethanol	Rose and honey aromas	9222 ± 864 ^a^	6631 ± 131 ^b^
G21	Naphthalene	Coal tar and camphor ball aromas	N.D.	13 ± 2
B1	Ethyl acetate	Fruity aroma	30,844 ± 755 ^a^	25,192 ± 328 ^b^
B2	Isoamyl acetate	Fruit aroma	N.D.	N.D.
B4	Ethyl caproate	Fruit aroma	248 ± 76 ^a^	48 ± 16 ^b^
B9	Octanoic acid ethyl ester	Fruity and floral aromas	1795 ± 127 ^a^	628 ± 32 ^b^
B13	Decanoic acid ethyl ester	Coconut, fruit, and floral aromas	955 ± 14 ^a^	961 ± 11 ^a^
B29	Phenylethyl acetate	Rose and honey aromas	366 ± 61 ^a^	15 ± 4 ^b^
B30	Dodecanoic acid ethyl ester	Floral, fruity, and oily aromas	147 ± 18 ^a^	102 ± 9 ^b^
B31	Tetradecanoic acid ethyl ester	Coconut aroma	776 ± 23 ^a^	42 ± 10 ^b^
B35	Hexadecenoic acid ethyl ester	Cream and wax aromas	34 ± 9 ^a^	11 ± 3 ^b^

Note: Columns with different letters indicate significant differences (*p* < 0.05).

## Data Availability

The original contributions presented in the study are included in the article/Supplementary Material, further inquiries can be directed to the corresponding author.

## Supplementary Materials

The following supporting information can be downloaded at https://www.mdpi.com/article/10.3390/foods13193175/s1, Table S1: The information of sequencing data; Figure S1: Heat map of volatile flavor substances during fermentation in CK and SY. (A) Alcohols; (B) esters; (C) acids; Figure S2: Heat map of volatile flavor substances during fermentation in CK and SY. (D) Aldehydes; (E) ketones; (F) phenols; (G) hydrocarbons; (H) others; Figure S3: Heat map of volatile flavor substances with OAV > 1 during fermentation in SY and CK; Figure S4: Functional classification of microoraganisms based on KEGG pathway at level 1 in the fermentation; Figure S5: Functional classification of microoraganisms based on KEGG pathway at level 2 in the fermentation.
