# Supplementary material for: Effects of Saccharomyces cerevisiae and Cyberlindnera fabianii Inoculation on Rice-Flavor Baijiu Fermentation
Source: Foods. 2024 Oct 6;13(19):3175. doi: 10.3390/foods13193175 (PMC11476301; doi:10.3390/foods13193175)
Supplement: Supplementary file 1 [file foods-13-03175-s001.zip › foods-3206131-supplementary.pdf]

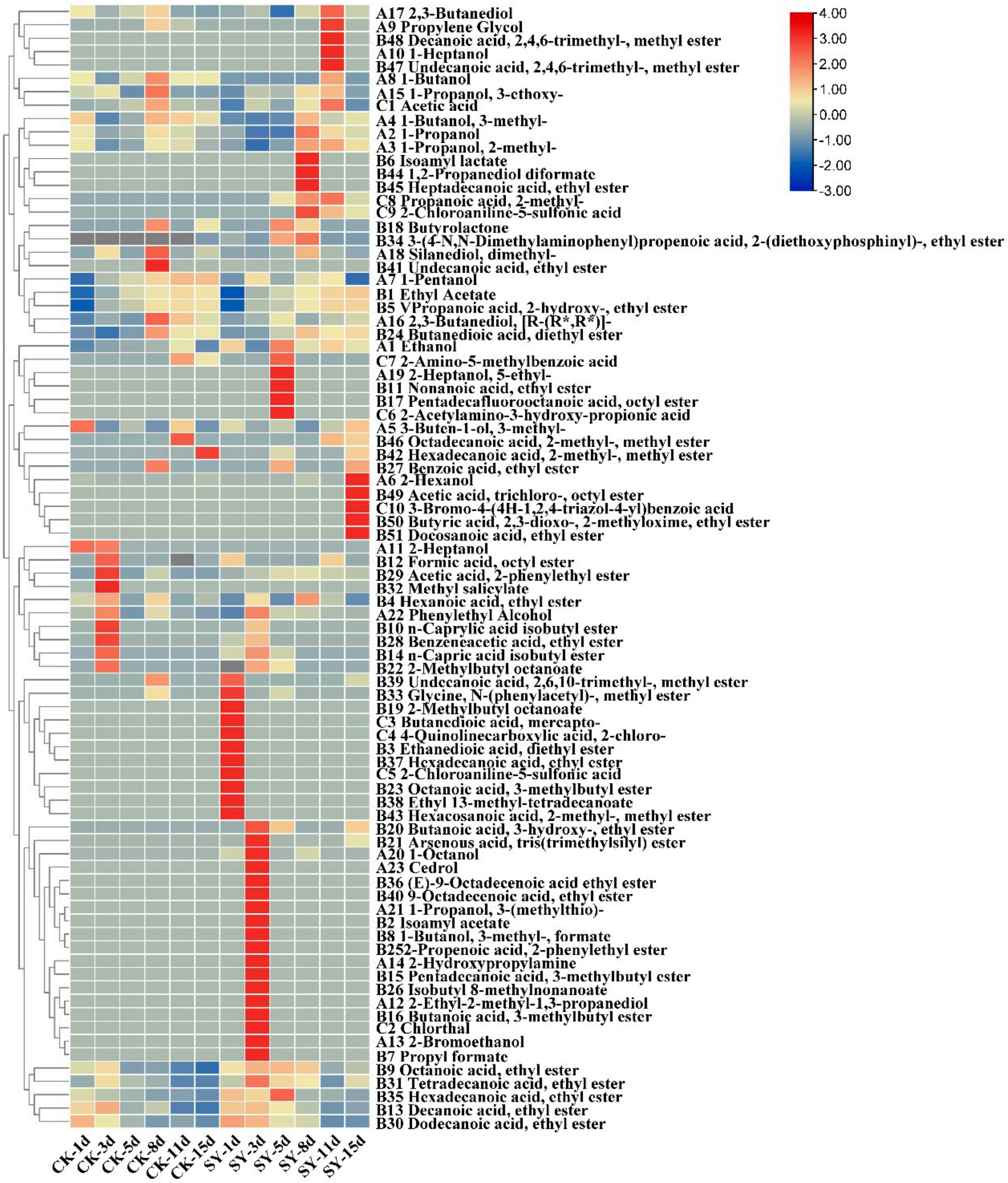

Figure S1. Heat map of volatile flavor substances during fermentation in CK and SY. (A) alcohols, (B) esters, (C) acids.

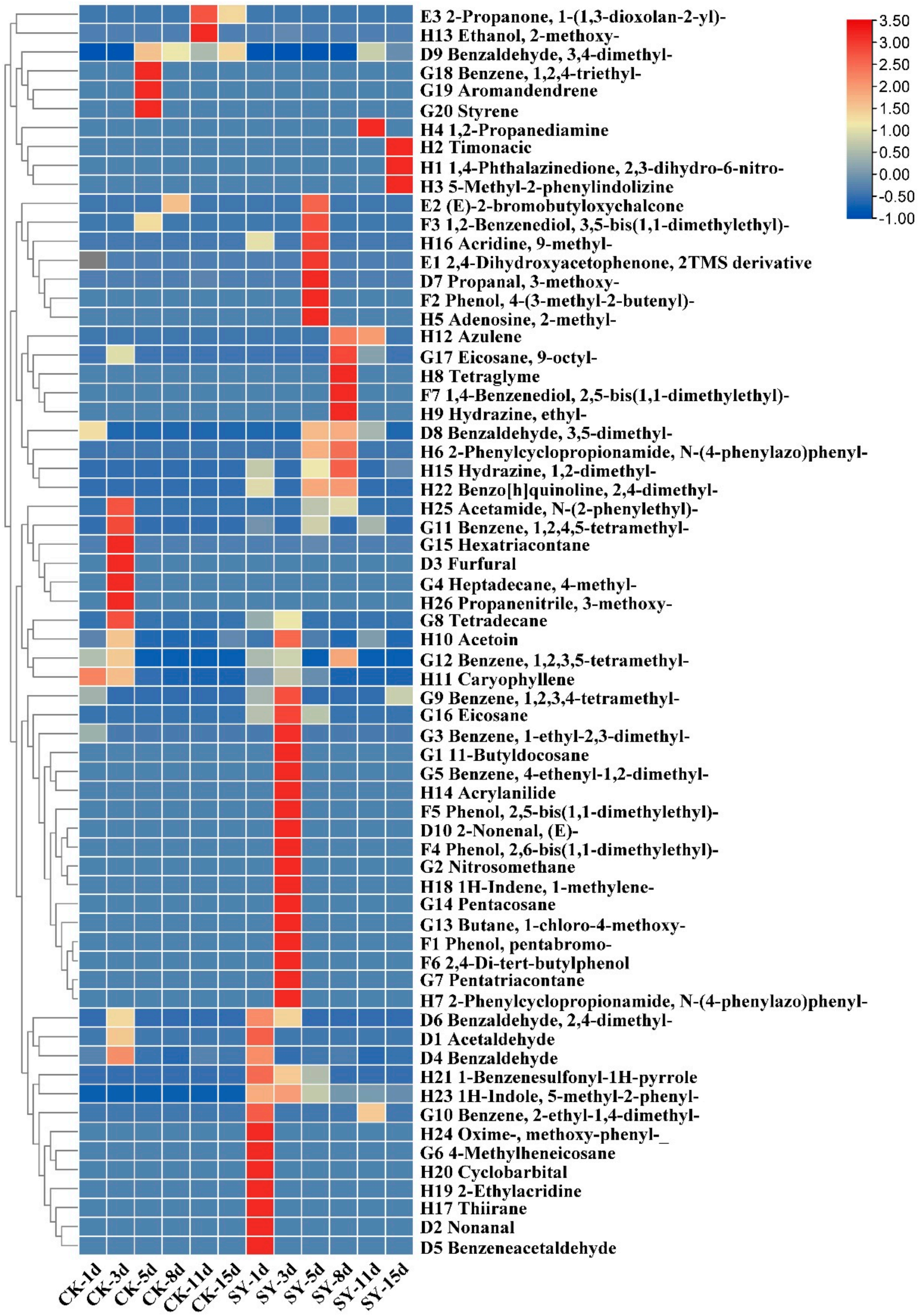

Figure S2. Heat map of volatile flavor substances during fermentation in CK and SY. (D) aldehydes, (E) ketones, (F) phenols, (G) hydrocarbons, (H) others.

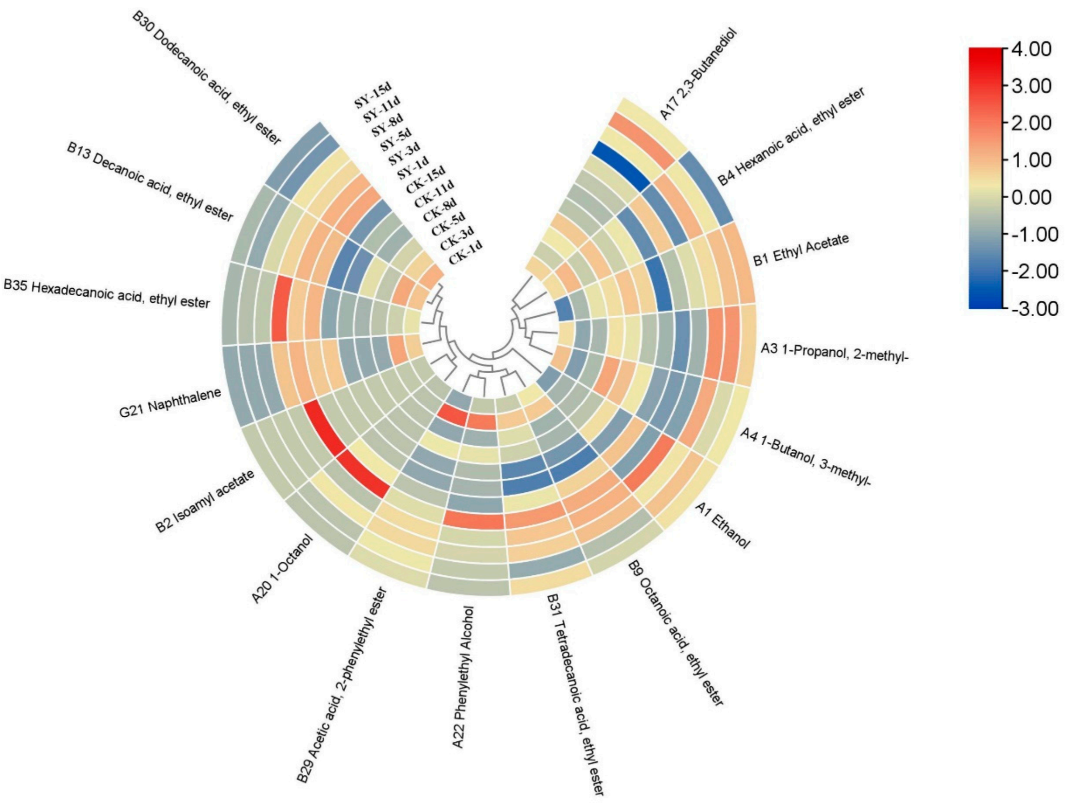

Figure S3. Heat map of volatile flavor substances with OAV > 1 during fermentation in SY and CK.

Table S1. The information of sequencing data.

Sample	Clean Data (Gbp)	Clean Q20 (%)	Clean Q30 (%)	Contigs Number	Average length（bp）	N50 (bp）	N90 (bp）	unigenes
CK3d-1	9.94	96.22	90.28	15183	5823.18	33054	1709	54966
CK3d-2	9.89	96.19	90.16	15283	5810.36	32014	1733	56095
CK11d-1	10.53	96.37	90.53	16869	5344.82	32461	1595	61530
CK11d-2	10.16	96.42	90.69	13427	6522.7	34471	1870	52073
SY3d-1	10.19	96.27	90.4	14510	6107.25	33596	1787	56316
SY3d-2	10.20	96.4	90.6	13354	6515.07	34959	1904	60450
SY11d-1	10.15	96.47	90.82	15572	5727.14	31530	1705	66508
SY11d-2	10.02	96.48	90.81	16131	5549.02	32743	1652	65298

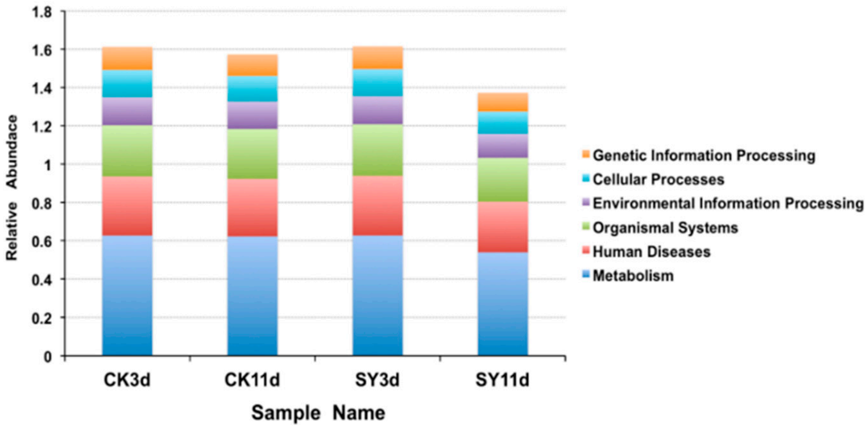

Figure S4. Functional classification of microoraganisms based on KEGG pathway at level 1 in the fermentation.

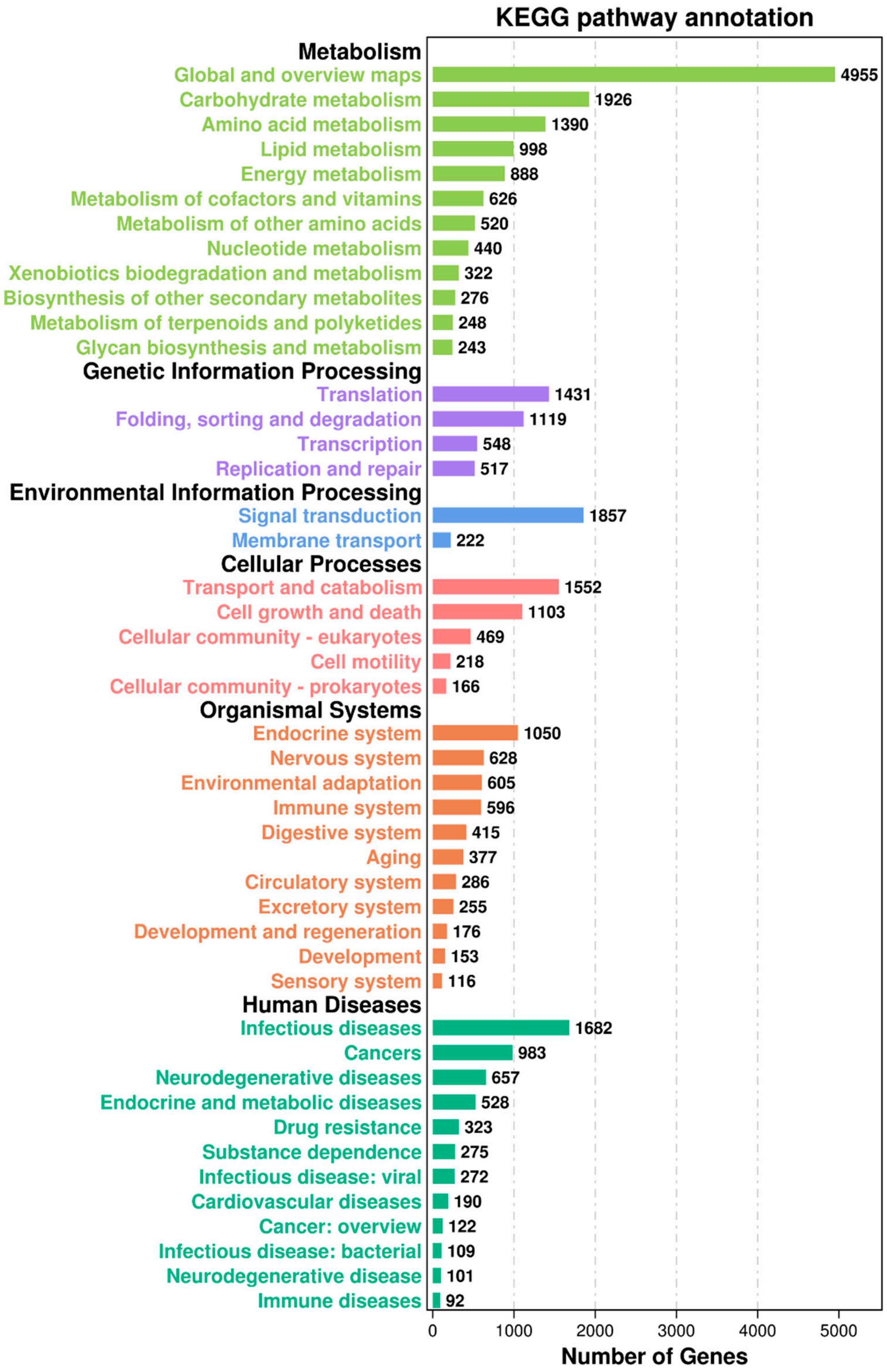

Figure S5. Functional classification of microoraganisms based on KEGG pathway at level 2 in the fermentation.
